# The Role of Ferritin and Folate in Determining Stem Cell Collection for Autologous Stem Cell Transplant in Multiple Myeloma

**DOI:** 10.3390/hematolrep17010005

**Published:** 2025-01-24

**Authors:** Charles J. Weeks, Mohammad Mian, Michael Stokes, Matthew Gold, Anvay Shah, Rohan Vuppala, Katherine J. Kim, Abigayle B. Simon, Jorge Cortes, Anand Jillela, Vamsi Kota

**Affiliations:** 1 Medical College of Georgia, Augusta University, Augusta, GA 30912, USA; chweeks@augusta.edu (C.J.W.); micstokes@augusta.edu (M.S.); mgold@augusta.edu (M.G.); anvshah@augusta.edu (A.S.); rvuppala@augusta.edu (R.V.); katkim@augusta.edu (K.J.K.); absimon@augusta.edu (A.B.S.); 2 Georgia Cancer Center, Augusta University, Augusta, GA 30912, USA; mmian@augusta.edu (M.M.); jorge.cortes@augusta.edu (J.C.); ajillella@augusta.edu (A.J.)

**Keywords:** multiple myeloma, autologous transplantation, hematopoietic stem cell mobilization, ferritin, folic acid, hemoglobin

## Abstract

Background: An autologous stem cell transplant (ASCT) is the standard of care for eligible patients with multiple myeloma (MM). However, the success of ASCT largely hinges on efficient mobilization; thus, a thorough analysis of factors that may affect mobilization is essential. Methods: The study consists of a single-center, retrospective chart review of 292 adult patients undergoing their first or second autologous transplantation for MM from 2016 to 2023. Patient demographics, serum lab values at the pre-collection evaluation visit, total stem cell capture (TC) in CD34/kg × 10^6^ stem cell capture on the first day of apheresis (FC) in CD34/kg × 10^6^, and the total number of days of apheresis (DOA) were retrieved from the electronic medical record (EMR). Results: Individuals with high folate levels experienced less DOA (1.43 ± 0.61) compared to those with normal folate levels (1.68 ± 0.82, *p* = 0.013). The high-folate group had a greater FC (3.26 ± 1.07) compared to the normal-folate group (2.88 ± 1.13, *p* = 0.013). High ferritin levels were associated with more DOA (1.79 ± 0.89) compared to the normal-ferritin group (1.51 ± 0.67, *p* = 0.034). Moderate anemia was significantly associated with decreased FC (*p* = 0.023) and increased DOA (*p* = 0.030). Abnormal hemoglobin (Hgb), ferritin, and folate statuses did not exhibit significant differences in survival analysis. Conclusions: The findings reveal that folate, ferritin, and Hgb levels are significantly associated with apheresis outcomes, offering guidance for optimizing stem cell mobilization in patients with MM.

## 1. Introduction

Multiple myeloma (MM) is characterized by the uncontrolled proliferation of malignant plasma cells within the bone marrow, which typically leads to osteolytic bone lesions, anemia, hypercalcemia, and renal insufficiency [1,2]. Each year in the United States, more than 35,000 new MM cases are diagnosed, and 12,500 patients die due to the disease, with the overall five-year relative survival rate being 61.1% [3]. While monoclonal antibodies have been introduced in the last five years as a promising option, autologous stem cell transplantation (ASCT) following high-dose chemotherapy remains the cornerstone of treatment, especially in eligible standard-risk patients [4]. ASCT involves the collection and reinfusion of a patient’s own hematopoietic stem cells and has become the standard of care for eligible multiple myeloma patients, offering a chance for durable remission [5].

The success of ASCT largely hinges on the efficient mobilization of peripheral blood stem cells (PBSCs), as this harvested cell population serves as the foundation for reconstitution of the hematopoietic system following transplantation. The mobilization of these stem cells, primarily achieved through the administration of granulocyte colony-stimulating factors (G-CSFs), is a critical step in the success of PBSC harvest [4]. In combination with the efficacy of G-CSF administration, special consideration is also given to anemia status, platelet count, and certain nutritional deficiencies, including folate, vitamin B12, and ferritin, because of their potential to affect the mobilization process [6,7,8,9].

The current study delves into the significance of ferritin and folate as a prognostic marker for the assessment of stem cell mobilization in MM patients undergoing ASCT. Both ferritin and folate are routinely evaluated in the workup of patients undergoing ASCT and may serve as a valuable tool in the stratification of patients at risk for impaired stem cell mobilization. This study aims to shed light on the use of serum biomarkers in predicting the efficacy of stem cell mobilization in the context of patients with MM undergoing transplantation.

## 2. Methods

### 2.1. Patients

We conducted a retrospective chart review of 306 patients that underwent an ASCT for multiple myeloma at the Georgia Cancer Center from May 2016 to May 2023. Patients (*n* = 14) missing ferritin labs at pre-collection or stem cell capture data in the electronic medical record (EMR) were excluded from the analysis. Thus, the total study cohort consisted of 292 patients. Patient demographics, serum lab values at the pre-collection evaluation visit, plerixafor use, total stem cell capture (TC), stem cell capture on the first day of apheresis (FC), the total number of days of apheresis (DOA), time to myeloid engraftment, and time to platelet engraftment were retrieved from the EMR. Pre-collection was defined as the last visit before patients underwent mobilization and apheresis, in which they had labs drawn and were evaluated for progression eligibility. Patients were not instructed to take and were not given vitamin supplements, and patient-reported data on home vitamin supplementation were not collected.

### 2.2. Mobilization

Per institution protocol, mobilization was initiated by 10 μg/kg of G-CSF, administered subcutaneously each morning for four days and continued the following days during apheresis [10]. On the fourth day and each day after, the peripheral blood (PB) CD34-positive cells were enumerated. If the patient had a PB CD34 count of less than 20 or 40 CD34 cells/μL on the fourth or fifth day, respectively, plerixafor was scheduled each night [11]. Apheresis began on the fifth day and was continued each day until 3–4 CD34 cells/kg were collected. If apheresis required more than 4 days, the mobilization was considered a failure. None of the patients in this cohort had a mobilization failure. Myeloid engraftment was defined as an absolute neutrophil count (ANC) of ≥500/µL, while platelet engraftment was defined as independence from platelet transfusion for at least 7 days with a platelet count of >20 × 10^9^/L. After the transplantation, G-CSF was administered on day 5 and every day afterward until myeloid engraftment was reached.

### 2.3. Clinical Laboratory Values

The labs reported all came from the Wellstar-MCG hospital lab from 2016 to 2023 and were unchanged during this period. Ferritin levels having a serum concentration less than or equal to 50 ng/mL were classified as low [12], with normal being between 50.1 and 500 ng/mL and high being greater than 500 ng/mL [13,14]. A normal folate concentration was defined as 2.7–17.0 ng/mL [15], a normal vitamin B12 concentration was defined as 200–900 pg/mL [16,17], and a normal platelet count was defined as 150–400 platelets times 1000/mL [18]. Hemoglobin (Hgb) concentration served as the criterion for defining anemia grades. For women, a Hgb level above 12 g/dL was classified as normal, while for men, the threshold was above 13.5 g/dL. Mild anemia was categorized as Hgb levels between 12 and 10 g/dL for women and between 13.5 and 10 g/dL for men. Moderate anemia was defined for levels between 8 and 10 g/dL, and severe anemia was classified as levels below 8 g/dL [19,20]. Only 2 patients had severe anemia, so it was not analyzed as a group.

### 2.4. Statistical Analysis

Continuous variables were summarized using means and standard deviations (mean ± SD) and compared across serum concentration categories using a one-way analysis of variance (ANOVA). Pearson’s correlation was used to assess for associations between sex and anemia grade. The categorical variables were presented as frequencies and percentages, and the group comparisons were performed using the Pearson’s chi-square test. The subjects were analyzed for basic demographics, apheresis variables, and serum lab concentrations. Models were adjusted for potential confounders, including age, race, and Hgb concentration. A survival analysis was run as a Kaplan–Meier estimate. A statistical analysis was run using IBM SPSS Statistics, version 29.0.0.0, and GraphPad Prism, version 10.

## 3. Results

### 3.1. Baseline Demographics, Platelet Counts, Anemia, and B12

**Figure 1:**
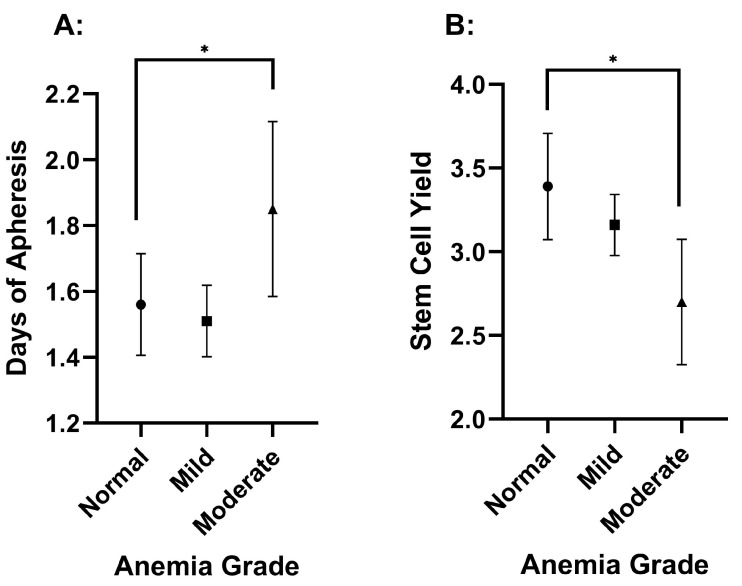
Hemoglobin level effect on the duration of apheresis (**A**) and stem cell yield (**B**). The values are presented as means, with error bars as the 95% confidence interval. Stem cell yield is in CD34/CD34/kg × 10^6^. Significance is indicated by an asterisk and has a *p*-value of less than 0.05.

The study cohort consisted of 292 patients. Of these, 162 (55.5%) were male and 130 (44.5%) were female. The racial composition of the cohort was 42.5% White (*n* = 124), 56.5% Black (*n* = 165), and 1.0% categorized as Other (*n* = 3). The mean age of the participants was 61.3 years (±10.2). The mean serum folate concentration was 16.9 ng/mL (±10.4), and the mean serum vitamin B12 concentration was 696 pg/mL (±1035). The mean Hgb concentration was 11.9 g/dL (±1.6), and the mean platelet count was 227 × 1000/mL (±74). In terms of Hgb concentration, moderate anemia had a significantly lower FC (*p* = 0.023) and a longer DOA (*p* = 0.030) than patients without anemia or with mild anemia (Figure 1 and Table 1). Anemia grade continued to have a significant effect on FC (*p* = 0.021) and DOA (*p* = 0.040), even after covarying for sex. The anemia grade was not significantly different between sexes (*p* = 0.099), and there was no correlation between sex and DOA (*p* = 0.097) or FC (*p* = 0.197). Also, there was no significant difference between anemia grades with respect to plerixafor usage (*p* = 0.947). Platelet count and serum B12 concentration did not have a significant effect on DOA or FC (Table 1).

### 3.2. Differences Among Pre-Collection Folate Serum Concentrations

The sex distribution did not reveal a significant association with folate levels (*p* = 0.676), and there was no significant difference in race between the two groups (*p* = 0.306). Age was statistically significant between the two groups, with the high-folate group having a higher (*p* = 0.039) mean age (63.6 ± 9.7 years) compared to the normal-folate group (60.9 ± 9.5 years). Notably, Hgb levels also exhibited a significant difference, with the high-folate group showing higher Hgb concentrations (12.1 ± 1.6) in comparison to the normal-folate group (11.6 ± 1.8, *p* = 0.019). Ferritin concentration, B12 concentration, plerixafor use, and platelet count were not significantly different between the folate groups (Table 2).

**Figure 2:**
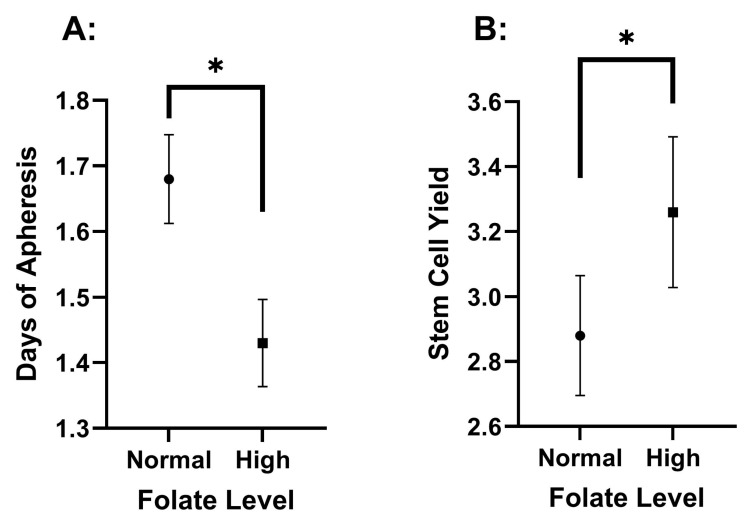
Folate level effect on the duration of apheresis (**A**) and stem cell yield (**B**). The values are presented as means, with error bars as the 95% confidence interval. Stem cell yield is in CD34/CD34/kg × 10^6^. Significance determined by ANOVA, indicated by an asterisk, and has a *p*-value less than 0.05.

Folate levels showed a statistically significant difference in DOA across the high and normal concentration categories. Specifically, the normal-folate group exhibited a significantly higher DOA (1.68 ± 0.82) than the high-folate group (1.43 ± 0.61, *p* = 0.013). The high-folate group had a significantly higher FC (3.26 ± 1.07) than the normal-folate group (2.88 ± 1.13, *p* = 0.013) (Figure 2 and Table 1). These differences for FC (*p* = 0.028) and DOA (*p* = 0.039) remained statistically significant even after adjusting for age and Hgb levels. Notably, TC also exhibited no significant differences between the two folate groups (Supplemental Table S1).

### 3.3. Differences Among Pre-Collection Ferritin Serum Concentrations

There was a significant difference between race and ferritin level (*p* = 0.002) but not between genders (*p* = 0.206). The high-ferritin group exhibited a higher proportion of Black individuals than the normal- and low-ferritin groups, suggesting a potential racial disparity. Moreover, the study revealed a significant difference in Hgb concentrations among ferritin groups. The high-ferritin group had significantly lower Hgb levels than the normal- and low-ferritin groups (*p* < 0.001), highlighting the association between ferritin status and the severity of anemia in the study population. These differences were, therefore, used as covariates. Age, folate concentration, B12 concentration, plerixafor use, and platelet count were not significantly different between the groups (Table 3).

**Figure 3:**
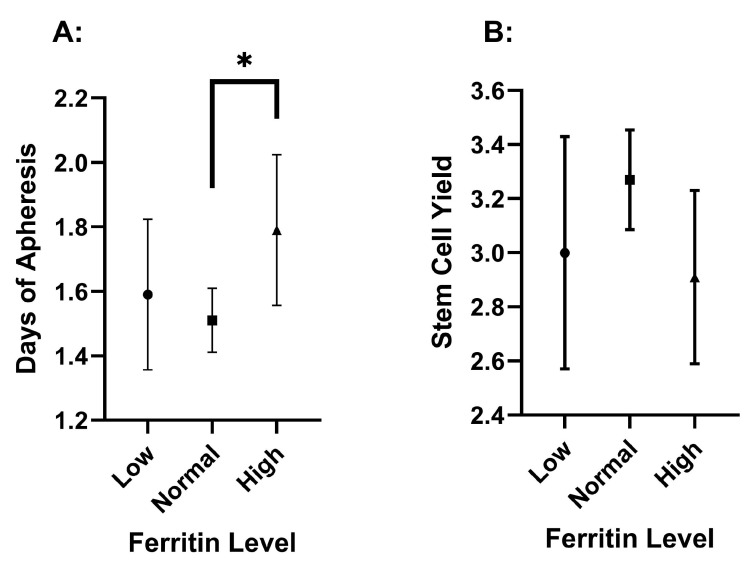
Ferritin level effect on the duration of apheresis (**A**) and stem cell yield (**B**). The values are presented as means, with error bars as the 95% confidence interval. Stem cell yield is in CD34/CD34/kg × 10^6^. Significance is indicated by an asterisk and has a *p*-value less than 0.05.

DOA was significantly different (*p* = 0.034) between ferritin levels, but the FC was not significantly different between them (Figure 3 and Table 1). Notably, the DOA demonstrated a statistically significant difference when comparing the normal- and high-ferritin groups. The high-ferritin group showed a significantly longer DOA (1.79 ± 0.89) compared to the normal-ferritin group (1.51 ± 0.67, *p* = 0.009) (Supplemental Table S2), with adjustment for race and Hgb. Also, the high-ferritin group (2.91 ± 1.22) nearly had a significantly lower FC compared to the normal group (3.27 ± 1.30, *p* = 0.064). Meanwhile, the low-ferritin group did not have significantly lower FC or greater DOA compared to the normal group. The total stem cell capture (TC) was never significantly different between any of the groups (Supplemental Table S2).

### 3.4. Survival and Engraftment Differences Among Pre-Collection Ferritin and Folate Concentrations

Survival analyses were run on this cohort with a median follow-up of 2.48 years and a standard deviation of 1.46 years. However, although there were significant differences in the collection for the different serum concentrations of folate and ferritin, there were no significant differences in survival between different ferritin (*p* = 0.469), folate (*p* = 0.823), and Hgb (*p* = 0.445) levels (Supplemental Figure S1). There were also no significant differences in the likelihood of disease progression for the differences in ferritin (*p* = 0.499), folate (*p* = 0.165), and Hgb (*p* = 0.614) status at pre-collection (Supplemental Figures S2–S4).

**Figure 4:**
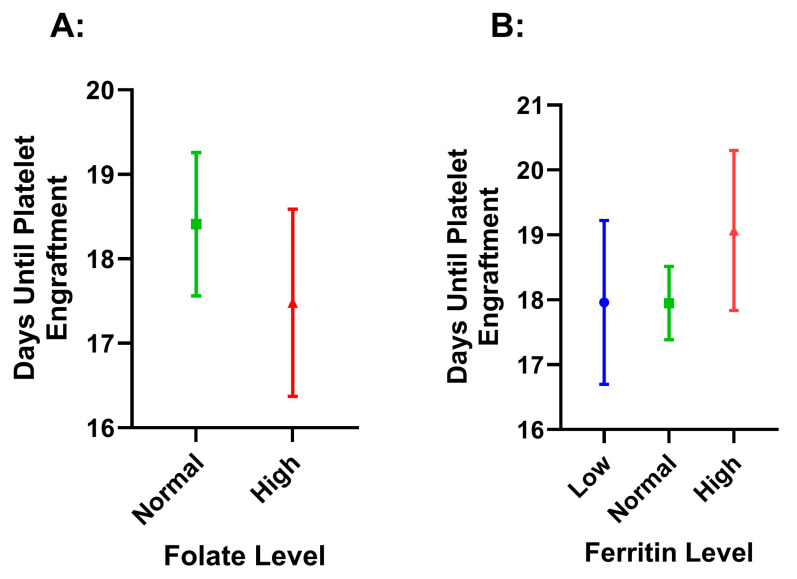
Time to platelet engraftment by folate (**A**) and ferritin (**B**) level. The values are presented as means, with error bars as the 95% confidence interval. Low = ferritin level < 50 ng/mL, normal = ferritin level between 50.1 and 500 ng/mL, and high = ferritin level > 500 ng/mL. Normal folate was considered as 2.7 to 17.0 ng/mL, with high being considered as anything greater than 17 ng/mL. The number of days was counted from the time of transplant until platelet recovery was recorded.

**Figure 5:**
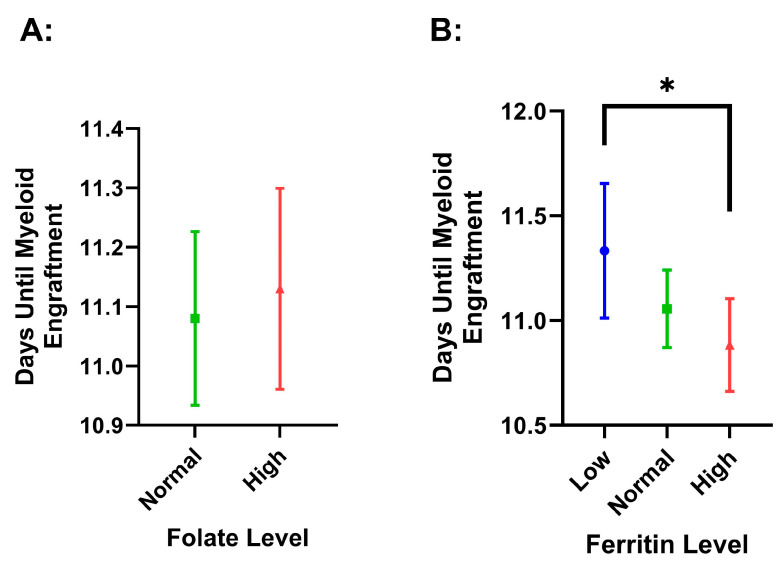
Time to myeloid engraftment via ANC count recovery by folate (**A**) and ferritin (**B**) level. The values are presented as means, with error bars as the 95% confidence interval. Low = ferritin level < 50 ng/mL, normal = ferritin level between 50.1 and 500 ng/mL, and high = ferritin level > 500 ng/mL. Normal folate was considered as 2.7 to 17.0 ng/mL, with high being considered as anything greater than 17 ng/mL. None of the patients had low folate. The number of days was counted from the time of transplant until ANC recovery was recorded. Significance is indicated by an asterisk and has a *p*-value less than 0.05.

Folate status did not have a significant effect on platelet (*p* = 0.196) or myeloid (*p* = 0.680) time to engraftment, and we did not find differences with respect to normal or high serum folate status for time to platelet engraftment (*p* = 0.168) (Figure 4). We did find that there was a significant difference between low ferritin (11.33 ± 1.24) and high ferritin (10.88 ± 0.97, *p* = 0.019) for days to myeloid engraftment. However, this difference did not maintain significance after covarying for age and Hgb (*p* = 0.118) (Figure 5).

## 4. Discussion

ASCT is the standard of care for MM, and the success of ASCT largely hinges on efficient mobilization. The present study aimed to investigate the impact of pre-collection serum concentrations of various biomarkers, including folate, vitamin B12, ferritin, Hgb, and platelet count, on the outcomes of stem cell mobilization in patients with MM preparing for ASCT. The current analysis revealed that folate, ferritin, and hemoglobin levels are significantly associated with mobilization outcomes in patients with MM.

### 4.1. The Association of Elevated Folate with Improved Mobilization

Higher folate concentrations are being associated with a shorter DOA and a greater FC. Specifically, individuals with normal folate levels exhibited a longer DOA compared to those with high folate levels (*p* = 0.013). In contrast, the high-folate group had a significantly higher FC compared to the normal-folate group (*p* = 0.013). Notably, the TC was not significantly different between the folate groups, suggesting that the increased DOA was not due to excessive stem cell capture, emphasizing the clinical significance of the longer duration.

Although the high-folate group was older and had higher Hgb, other factors, such as race, sex, ferritin concentration, B12 concentration, and platelet count, did not significantly differ between the normal- and high-folate groups. Also, the statistically significant differences between folate groups for DOA and FC remained after covarying for Hgb and age, emphasizing the specificity of the relationship.

Folate is well studied for its roles in one-carbon metabolism, which plays a role in DNA synthesis [21]. At present, it is uncertain precisely how it is relevant to the mobilization of peripheral stem cells. The impact of folic acid on increasing hematopoiesis is well described, which could theoretically decrease the pool of available stem cells [22]. However, it has also been previously shown that the short-term administration of high levels of folate increases the committed progenitor cell count [23]; thus, high levels may play a significant role in apheresis efficiency.

The current findings suggest that individuals with high folate levels may require fewer days of apheresis and enhanced early stem cell capture, so physicians can anticipate better mobilization in this population, thus indicating a potential need for a shift in clinical practice. These findings suggest that a high folate level may benefit stem cell mobilization and call for future studies as to whether pre-collection folate supplementation could improve apheresis efficiency.

### 4.2. The Association of Abnormal Ferritin with Poorer Mobilization

Low ferritin has proposed negative implications on the efficacy of peripheral stem cell mobilization [6,9], but this study did not find a statistically significant association between low ferritin and worse apheresis outcomes. Iron metabolism has become a topic of significant discussion, with emerging hypotheses suggesting that anemia could worsen in individuals with iron deficiency due to the combined effects of G-CSF and erythropoiesis inhibition. Specifically, the increase in erythropoietin levels, triggered by iron deficiency, may drive the differentiation of stem cells into erythroid progenitor cells, ultimately reducing the pool of viable stem cells available for collection [9]. Several studies have shown that iron deficiency is a negative prognostic indicator for the success of the stem cell mobilization process in allogeneic donors [6,9]. However, an in vitro colony-forming unit assay, a common predictor of engraftment, performed on peripheral blood samples from a pediatric donor population, indicated that iron deficiency had no effect on the peripheral stem cell potencies [24].

Iron overload, on the other hand, may occur in this particular population due to the initial cancer therapy, where patients are often subjected to multiple RBC transfusions [25]. Multiple studies have indicated that higher ferritin levels are associated with increased mortality prior to and following ASCT due to higher incidence of organ toxicity, infection, and acute graft-versus-host disease (GVHD), which makes successful ASCT less likely [26,27,28]. Previous studies have shown that patients with iron overload have significantly lower mobilized CD34+ cell concentrations for autologous transplantation in peripheral blood than those with low ferritin in patients with various hematological malignancies [29] and AML [30]. Also, allogeneic transplant studies have shown similar results, where high ferritin was associated with poor mobilization of peripheral stem cells, reduced engraftment, and increased complications following transplantation [6,9]. Those results indicate that iron overload is an independent prognostic variable for decreased mobilization potential, but this phenomenon has not previously been shown in a substantial myeloma patient group.

The current study highlights the influence of ferritin levels on the duration of apheresis procedures. The high-ferritin group displayed a longer DOA compared to the normal-ferritin group (*p* = 0.009). This extended duration may reflect the need for extended stem cell collection in people with elevated ferritin levels. Although not statistically significant, high ferritin may have a lower FC as well. On the other hand, low ferritin levels did not show a significantly different FC or DOA, arguing against collection delays in patients with relative iron deficiency. This finding is important because delaying stem cell collection can have many implications, including risk of infection, psychological stress, increased cost, and logistical challenges.

Race and hemoglobin concentration emerged as significant factors between the grouped ferritin levels. The high-ferritin group exhibited a higher proportion of Black individuals, suggesting potential racial disparities in ferritin concentrations. The high-ferritin group also had significantly lower Hgb levels compared to the normal-ferritin group, which is suggestive of anemia in the setting of an inflammatory state. However, the statistical significance for increased DOA in the high-ferritin group remained after covarying for race and Hgb, which suggests that high ferritin is independently indicative of more DOA.

### 4.3. Other Factors That May Influence Mobilization

The current analysis revealed the impact of hemoglobin concentration on stem cell apheresis. Individuals with moderate anemia had significantly lower FC (*p* = 0.023) and longer DOA (*p* = 0.030). Mild anemia did not significantly impair stem cell collection, so physicians may not need to anticipate impaired stem cell mobilization unless anemia is moderate. Many patients with MM preparing for a transplant have various grades of anemia, so this is crucial information for anticipating their mobilization success and aligns with previous research [31]. In response to this, plerixafor remains a viable pre-emptive or rescue therapy in the case of poor mobilization [32].

The megakaryopoeitic commitment to the totipotent stem cell could be a causative reason for the impact of platelet counts on the success of mobilization [33]. Because of this, platelet count remains a well-studied predictive factor for the success of peripheral stem cell mobilization, whereas thrombocytopenia is often associated with poor collection [26,34,35,36]. The current study, however, did not find an association between thrombocytopenia and poor collection in this specific myeloma cohort.

### 4.4. Engraftment and Survival

Folate and ferritin status did not appear to have significant associations with time to engraftment or overall survival. Myeloid and platelet engraftment were not significantly altered by folate or ferritin levels at the pre-collection visit after covariance. Therefore, engraftment and survival may be more heavily impacted by changes occurring during the conditioning therapy for a transplant. Also, we did not see significant differences between folate, Hgb, or ferritin levels in the overall survival or time to progression, which may be attributed to the lack of disease progression over our relatively short median follow-up of 2.48 years, as the national 5-year OS of patients transplanted after 2014 is expected to be 70% [37].

### 4.5. Limitations

This study has several limitations that should be considered when interpreting its findings. First, the study does not account for whether patients were undergoing their first or second ASCT or if they had required previous transfusions, which may impact mobilization outcomes. The study does not examine specific characteristics of myeloma, such as disease stage or treatment-related factors, such as prior chemotherapy or other interventions, which could have influenced patients’ hematologic parameters and stem cell mobilization. As our study focused solely on MM patients undergoing ASCT, we did not have a healthy control group with which to compare. Additionally, cytogenetic profiles were not evaluated, limiting insights into the influence of specific genetic abnormalities on mobilization success. Data on vitamin supplementation were not systematically collected, which limits our ability to analyze the impact of nutritional factors on the clinical outcomes observed. This study’s single-center, retrospective design may limit generalizability, as it reflects the protocols and patient characteristics unique to one institution. Finally, the short follow-up period also limits the ability to assess long-term survival outcomes and disease progression in this cohort.

## 5. Conclusions

The present findings provide valuable insights into the complex relationships between serum biomarkers, demographic factors, and stem cell collection. We found that folate, ferritin, and hemoglobin levels are key factors associated with the duration and initial collection of stem cells during mobilization for ASCT in patients with MM.

Individuals with high folate levels may require fewer days of apheresis and enhanced early stem cell capture, so physicians can anticipate better mobilization in this population. Ferritin and hemoglobin levels are additional factors influencing the duration of apheresis. The high-ferritin group required many more days of apheresis, while individuals with moderate anemia had significantly early stem cell capture and required more days of apheresis.

These findings may guide physicians in anticipating a patient’s mobilization and, ultimately, enhance the care and outcomes of patients with MM. However, further research is warranted to fully understand the underlying mechanisms and potential clinical applications of these findings. Likewise, future trials evaluating the effect of high-dose folate supplementation in stem cell mobilization may be warranted.

## Figures and Tables

**Table 1:** Descriptive values of pre-collection serum concentration on collection outcome.

	Low	Normal	High	*p*-Value
**Folate**	*n* = 0	*n* = 146	*n* = 84	
DOA		1.68 ± 0.82	1.43 ± 0.61	0.013 *
FC		2.88 ± 1.13	3.26 ± 1.07	0.013 *
**B12**	*n* = 23	*n* = 184	*n* = 45	
DOA	1.48 ± 0.73	1.61 ± 0.76	1.58 ± 0.78	0.735
FC	2.84 ± 1.29	3.41 ± 1.66	3.41 ± 1.66	0.151
**Platelet**	*n* = 38	*n* = 246	*n* = 8	
DOA	1.68 ± 0.70	1.56 ± 0.74	1.50 ± 0.76	0.605
FC	2.89 ± 1.19	3.20 ± 1.30	3.31 ± 1.63	0.364
**Ferritin**	*n* = 41	*n* = 193	*n* = 58	
DOA	1.59 ± 0.74	1.51 ± 0.70	1.79 ± 0.89	0.034 *
FC	3.00 ± 1.36	3.27 ± 1.30	2.91 ± 1.22	0.131
**Anemia Grade**	**Normal**	**Mild**	**Moderate**	***p*-Value**
	*n* = 83	*n* = 167	*n* = 40	
DOA	1.57 ± 0.74	1.51 ± 0.69	1.85 ± 0.83	0.030 *
FC	3.38 ± 1.51	3.17 ± 1.18	2.70 ± 1.17	0.023 *

Data are presented as mean ± standard deviation. FC = stem cell capture on the first day of apheresis in CD34/kg × (10^6^); DOA = total number of days of apheresis. * Indicates significance via ANOVA.

**Table 2:** Demographic and serum data from the pre-collection visit of the normal- and high-folate groups.

	Normal	High	*p*-Value
	*n* = 146	*n* = 84	
**Sex**			0.676
Male	81 (55.5%)	49 (58.3%)	
Female	65 (44.5%)	35 (41.7%)	
**Race**			0.306
White	55 (37.7%)	42 (50.0%)	
Black	90 (61.6%)	40 (47.6%)	
Other	1 (0.7%)	2 (2.4%)	
**Age**	60.9 ± 9.5	63.6 ± 9.7	0.039 *
**Ferritin**	341 ± 378	288 ± 350	0.288
**B12**	696 ± 1069	683 ± 754	0.928
**Hgb**	11.6 ± 1.8	12.1 ± 1.6	0.019 *
**Plerixafor**	1.12 ± 0.73	0.95 ± 0.74	0.082
**Platelet**	228 ± 76	228 ± 73	0.997

Data are presented as numbers and percentages of the total or as mean ± standard deviation. Age = age of the subject in years, Ferritin = serum concentration of ferritin in ng/mL, B12 = serum concentration of vitamin B12 in pg/mL, Hgb = serum concentration of Hgb in g/dL, Plerixafor = the number of plerixafor doses administered during mobilization, and Platelet = platelet count in number of platelets times 1000/mL. An asterisk indicates significance by ANOVA or chi-square analysis where appropriate.

**Table 3:** Demographic data and serum data from pre-collection visit of low-, normal-, and high-ferritin groups.

	Low	Normal	High	*p*-Value
	*n* = 41	*n* = 193	*n* = 58	
**Sex**				0.206
Male	21 (51.2%)	114 (59.1%)	27 (46.6.0%)	
Female	20 (48.8%)	79 (40.9%)	31 (53.4%)	
**Race**				0.002 *
White	18 (43.9%)	90 (46.6%)	16 (27.6%)	
Black	22 (53.7%)	103 (53.4%)	40 (69.0%)	
Other	1 (2.4%)	0 (0.0%)	2 (3.4%)	
**Age**	64.5 ± 11.5	60.7 ± 10.0	60.57 ± 9.4	0.066
**Ferritin**	17.9 ± 9.5	17.0 ± 10.6	14.8 ± 10.3	0.363
**B12**	668 ± 724	661 ± 954	871 ± 1301	0.426
**Hgb**	11.9 ± 1.5	12.1 ± 1.6	10.9 ± 1.8	<0.001 *
**Plerixafor**	1.17 ± 0.97	1.18 ± 0.75	1.14 ± 0.81	0.949
**Platelet**	241 ± 71	229 ± 75	213 ± 71	0.169

Data are presented as numbers and percentages of the total or as mean ± standard deviation. Age = age of the subject in years, Ferritin = serum concentration of ferritin in ng/mL, B12 = serum concentration of vitamin B12 in pg/mL, Hgb = serum concentration of Hgb in g/dL, Plerixafor = the number of plerixafor doses administered during mobilization, and Platelet = platelet count in number of platelets times 1000/mL. An asterisk indicates significance by ANOVA or chi-square analysis where appropriate.

## Data Availability

The original contributions presented in this study are included in the article/Supplementary Materials. Further inquiries can be directed to the corresponding author.

## Supplementary Materials

The following supporting information can be downloaded at https://www.mdpi.com/article/10.3390/hematolrep17010005/s1, Supplementary Table S1 shows the stem cell collection values of DOA, TC, and FC for the high- and normal-folate groups. Supplementary Table S2 shows the stem cell collection values of DOA, TC, and FC for the high-, normal-, and low-ferritin groups. Supplementary Figure S1 shows the Kaplan–Meier plots of survival probability by hemoglobin, ferritin, and folate level. Supplementary Figures S2–S4 show the probability of progression sorted by serum concentration groups of ferritin, hemoglobin, and folate, respectively.
