# Supplementary material for: The Role of Ferritin and Folate in Determining Stem Cell Collection for Autologous Stem Cell Transplant in Multiple Myeloma
Source: Hematol Rep. 2025 Jan 24;17(1):5. doi: 10.3390/hematolrep17010005 (PMC11855912; doi:10.3390/hematolrep17010005)
Supplement: Supplementary file 1 [file hematolrep-17-00005-s001.zip › hematolrep-3366306-supplementary.pdf]

A:

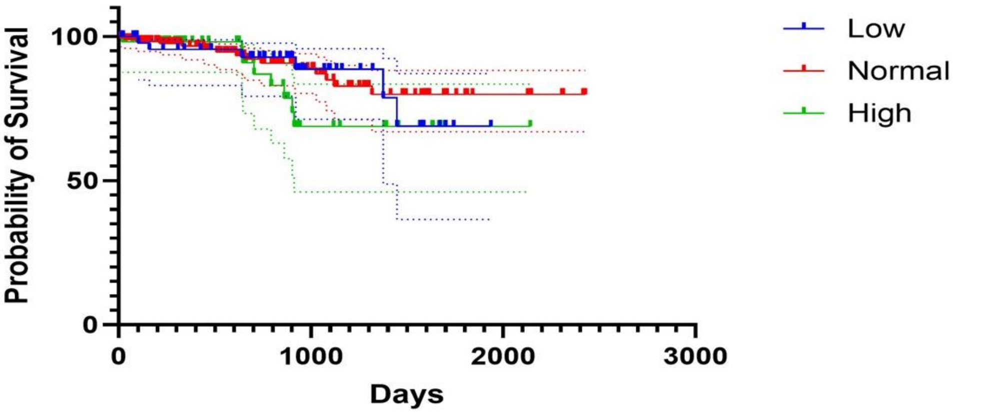

B:

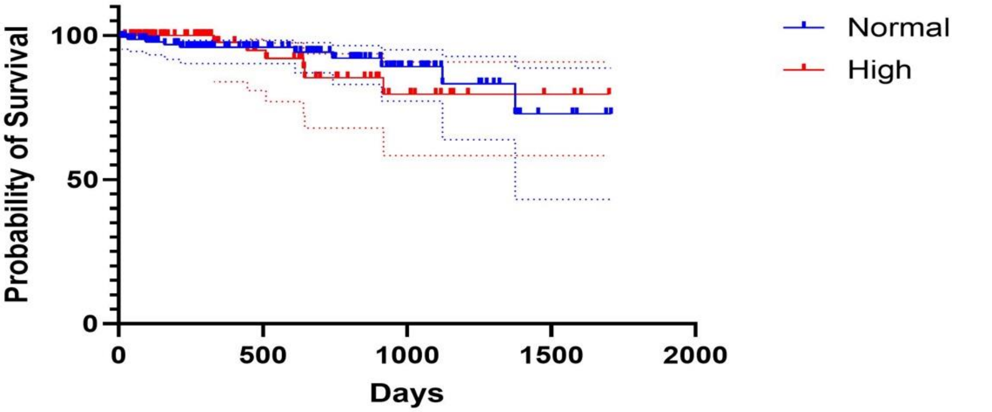

C:

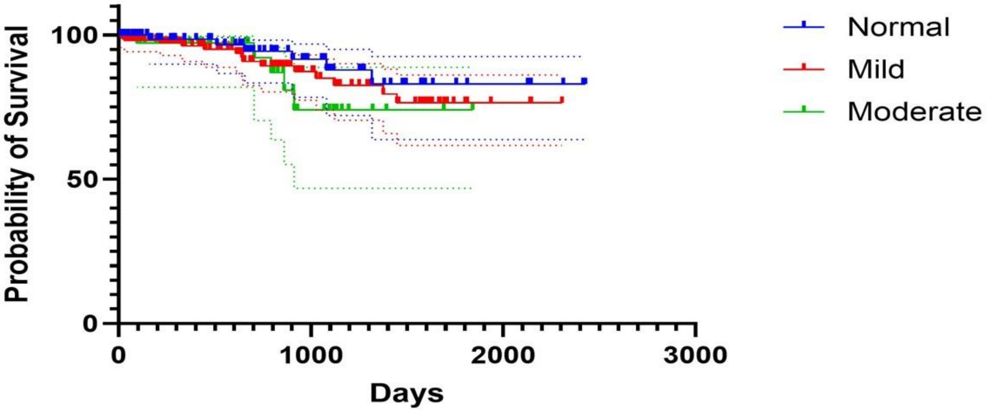

Supplemental Figure S1: The survival probability over time by (1A) ferritin, (1B) folate and (1C) hemoglobin level. Normal levels are >12 g/dL for women and >13.5 g/dL for men. Mild anemia ranges from 10 to 12 g/dL for women and 10 to 13.5 g/dL for men. Moderate anemia is defined as 8 to 10 g/dL, and severe anemia as <8 g/dL. Normal folate was considered as 2.7 to 17.0 ng/ml with high being considered as anything greater than 17 ng/ml. None of the patients had low folate. With respect to ferritin level, Low was < 50 ng/ml, normal was ferritin level between 50.1-500

ng/ml, and high was ferritin level > 500 ng/ml. The dotted lines represent 95% confidence intervals.

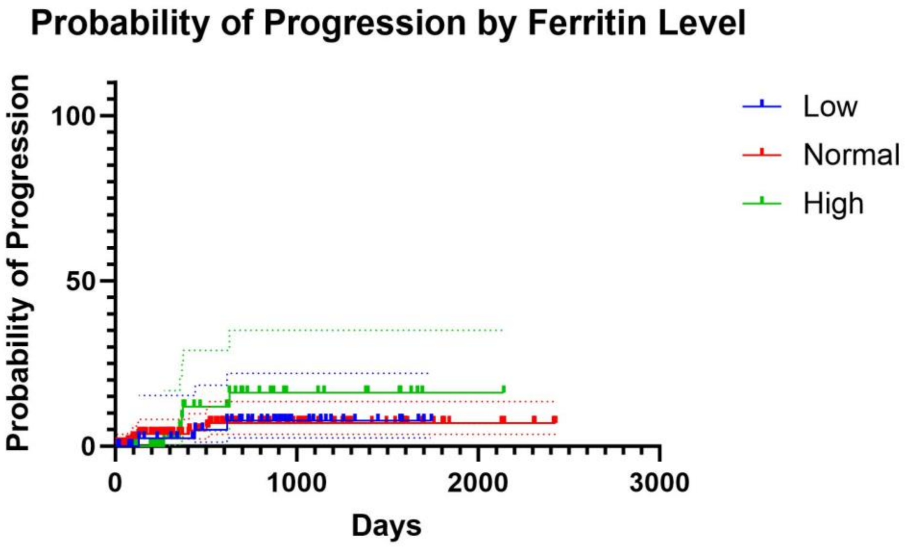

Supplemental Figure S2: The probability progression over time by ferritin level. Low = ferritin level < 50 ng/ml, Normal = ferritin level between 50.1-500 ng/ml, High = ferritin level > 500 ng/ml. The dotted lines represent 95% confidence intervals.

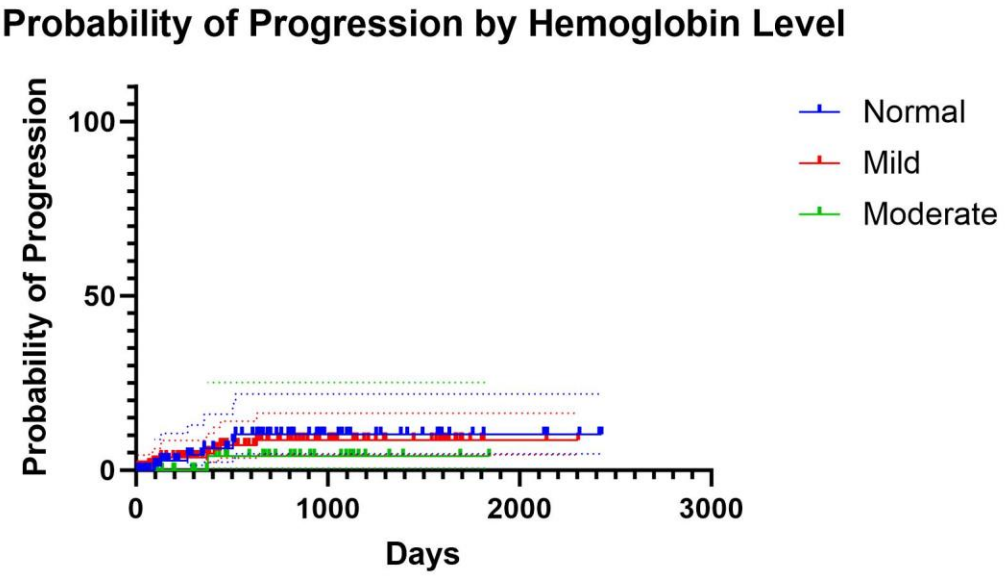

Supplemental Figure S3: The probability progression over time by anemia grade. Normal levels are >12 g/dL for women and >13.5 g/dL for men. Mild anemia ranges from 10 to 12 g/dL for women and 10 to 13.5 g/dL for men. Moderate anemia is defined as 8 to 10 g/dL, and severe anemia as <8 g/dL. The dotted lines represent 95% confidence intervals.

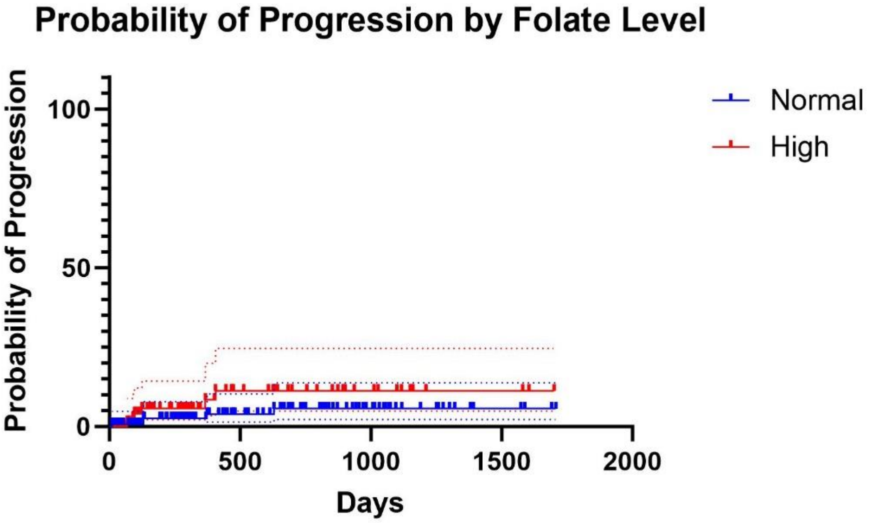

Supplemental Figure S4: The probability progression over time by folate level. Normal folate was considered as 2.7 to 17.0 ng/ml with high being considered as anything greater than 17 ng/ml. None of the patients had low folate.

Supplemental Table S1: Extended stem cell collection data of high and normal Folate groups

Variable	Folate Grouping		p-value
	**Normal (n=146)**	**High (n=84)**	
FC	2.88 ± 1.13	3.26 ± 1.07	0.028**
DOA	1.68 ± 0.82	1.43 ± 0.61	.039**
TC	3.91 ± 1.25	3.95 ± 1.17	0.820

Data are presented as Mean ± Standard Deviation. FC = Stem Cell Capture on the First Day of Apheresis in CD34/kg*(10^6), DOA = Total Number of Days of Apheresis, TC = Total Stem Cell Capture in CD34/kg*(10^6), Normal= folate serum concentration of 2.7 ng/ml to 17.0 ng/m, High= folate serum concentration > 17 ng/ml. ** Indicates significant by ANCOVA while covarying for age and Hgb.

Supplemental table S2: Stem Cell Collection Effects by Ferritin Level

Variable	Ferritin Grouping		p-value
	**Low (n=41)**	**Normal (n=193)**	
FC	3.00 ± 1.36	3.27 ± 1.30	0.240
DOA	1.59 ± 0.74	1.51 ± 0.67	0.509
TC	3.96 ± 1.12	4.06 ± 1.31	0.664
	**Normal (n=193)**	**High (n=58)**	
FC	3.27 ± 1.30	2.91 ± 1.22	0.064
DOA	1.51 ± 0.67	1.79 ± 0.89	0.009**
TC	4.06 ± 1.31	4.13 ± 1.35	0.693
	**Low (n=41)**	**High (n=58)**	
FC	3.00 ± 1.36	2.91 ± 1.22	0.727
DOA	1.59 ± 0.74	1.79 ± 0.89	0.225
TC	3.96 ± 1.12	4.13 ± 1.35	0.500

Data are presented as Mean ± Standard Deviation. FC = Stem Cell Capture on the First Day of Apheresis in CD34/kg*(10^6), DOA = Total Number of Days of Apheresis, TC = Total Stem Cell Capture in CD34/kg*(10^6), Low = ferritin level < 50 ng/ml, Normal = ferritin level between 50.1-500 ng/ml, High = ferritin level > 500 ng/ml. ** Indicates significant by ANCOVA while covarying for race and Hgb.
